# A dToF Ranging Sensor with Accurate Photon Detector Measurements for LiDAR Applications

**DOI:** 10.3390/s23063011

**Published:** 2023-03-10

**Authors:** Hengwei Yu, Long Wang, Jiqing Xu, Patrick Yin Chiang

**Affiliations:** 1 State Key Laboratory of ASIC & System, Fudan University, Shanghai 201203, China; 2 PhotonIC Technologies, Shanghai 201203, China

**Keywords:** ranging sensor, dToF, SPAD, MCU, matched filter, Center-of-Mass, precision, reflectance

## Abstract

Direct time-of-flight (dToF) ranging sensors based on single-photon avalanche diodes (SPADs) have been used as a prominent depth-sensing devices. Time-to-digital converters (TDCs) and histogram builders have become the standard for dToF sensors. However, one of the main current issues is the bin width of the histogram, which limits the accuracy of depth without TDC architecture modifications. SPAD-based light detection and ranging (LiDAR) systems require new methods to overcome their inherent drawbacks for accurate 3D ranging. In this work, we report an optimal matched filter to process the raw data of the histogram to obtain high-accuracy depth. This method is performed by feeding the raw data of the histogram into the different matched filters and using the Center-of-Mass (CoM) algorithm for depth extraction. Comparing the measurement results of different matched filters, the filter with the highest depth accuracy can be obtained. Finally, we implemented a dToF system-on-chip (SoC) ranging sensor. The sensor is made of a configurable array of 16 × 16 SPADs, a 940 nm vertical-cavity surface-emitting laser (VCSEL), an integrated VCSEL driver, and an embedded microcontroller unit (MCU) core to implement the best matched filter. To achieve suitably high reliability and low cost, the above-mentioned features are all packaged into one module for ranging. The system resulted in a precision of better than 5 mm within 6 m with 80% reflectance of the target, and had a precision better than 8 mm at a distance within 4 m with 18% reflectance of the target.

## 1. Introduction

The ranging sensor, one of the most precise techniques for 3D ranging and distance measurements, has attracted considerable attention in recent decades due to its extremely high performance and promising applications in automated systems interacting with the external environment, spanning across the consumer, industrial, and automotive fields [1,2]. Due to the fact that silicon single-photon avalanche diodes (SPADs) are solid-state detectors capable of single-photon sensitivity in the visible and near-infrared spectrum, which can be manufactured in complementary metal-oxide-semiconductor (CMOS) processes together with on-chip analog or digital circuitry, SPADs are the best candidates for 3D ranging applications [3,4]. In light detection and ranging (LiDAR) systems, the photon arrival time measured after a sufficient number of pulsed laser shots can be accumulated into a histogram, and the conventional method of distance extraction is the Center-of-Mass (CoM) algorithm [5]. However, the reflections can vary greatly depending on the reflecting surface shape and reflectivity. In real-world LiDAR systems, it is difficult to calculate depth accurately using the CoM method alone.

Various architectures have been proposed to achieve high accuracy ranging [6,7]. A basic approach to reduce the error of ranging is increasing the number of laser shots in the measurement, which can help to obtain a histogram distribution with a high signal-to-noise ratio (SNR), but lowers the frame rate [8]. A common approach to achieve high accuracy ranging is the use of scanning lasers, which illuminate only a single spot of the target scene at once [9]. In this technique, the field of view (FoV) of the laser source is much smaller compared with that of flash illumination, allowing a higher optical power, but scanning lasers are more expensive and more complex. Another approach is the use of the OR-Tree logic circuit to achieve pixel binning. The higher number of SPADs triggers the same time-to-digital converter (TDC), which helps improve SNR and reduces the error of ranging [10]. In this way, the higher the number of SPADs triggering the same TDC, the more timestamp information is lost.

To be able to extract accurate depth information, the signal of the histogram needs to be filtered with a best matched filter. In [11], in order to reduce the fluctuations in bin values due to shot-noise, the authors proposed a finite impulse response (FIR) filter prior to determining the locations of the ToF peaks. In [12], the authors reported that the filter is a normalized version of a histogram of hits collected by one SPAD in single-photon mode measuring the location of a target placed at a specific distance. After the filtering, even in the cases where the photon detection probability was quite low and the signal was almost covered by noise, the signal could be recovered by the filter method. In [13], an FIR filter was reported to improve accuracy by up to 2 times. In the state-of-the-art ranging sensor systems, the choice of filter used is effective for improving accuracy and filtering out noise. However, it is difficult to define the best matched filter for a real ranging system.

In this work, we design different types of matched filters to filter the histogram data in the real ranging system, and select the best matched filter to be integrated into the microcontroller unit (MCU) core. Firstly, we captured the data of histograms at different distances using a ranging sensor. Secondly, we defined different profile filters (pile-up, reverse pile-up, triangle, and square) to process the raw data of the histogram; the pile-up profile means the distortion in the second half of the histogram, and the reverse pile-up profile means the distortion in the first half of the histogram [14]. Finally, the depth extraction of the processed histogram data is performed using the CoM algorithm, the ranging standard deviation is calculated from multiple measurements of the distance at a specific location with different profile filters, and the filter with the best profile is selected as that with the smallest standard deviation. We implemented the best profile filter using an MCU core, and make an SoC system to perform ranging with different values of reflectivity. The system resulted in a precision of better than 5 mm within 6 m with 80% reflectance of the target, and a precision better than 8 mm at a distance within 4 m with 18% reflectance of the target.

This work is organized as follows: The ranging sensor chip implementation is described in Section 2. How to define the best profile-matched filter is described in Section 3. In Section 4, the best matched filter implemented in the MCU core and the real ranging system measurement results are introduced, and in Section 5, the discussion and conclusion are presented.

## 2. Ranging Sensor Chip Implementation

**Figure 1:**
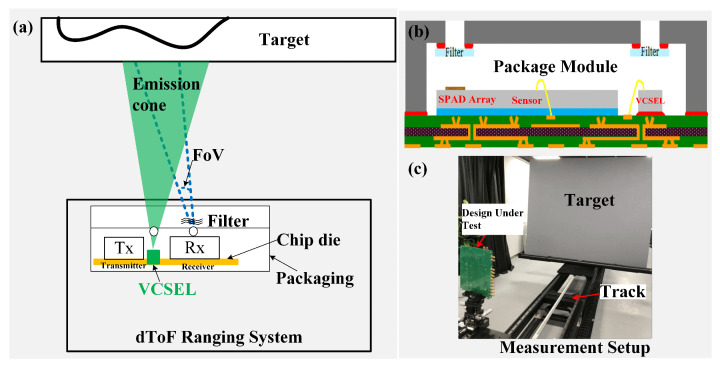
The designed dToF ranging system: (**a**) The working principle of dToF. (**b**) The package module of the ranging system. (**c**) The measurement setup of the ranging system.

We designed the dToF ranging system as shown in Figure 1. The dToF ranging system is designed to achieve highly accurate depth measurements, which requires the co-optimization of the hardware and firmware. Figure 1a illustrates the working principle of the ranging system, Figure 1b illustrates the package module of the ranging system, and Figure 1c illustrates the measurement setup of the ranging system. The chip was packaged into a module for ranging, the resulting package created an emission cone from a vertical-cavity surface-emitting laser (VCSEL) light source of 940 nm, which reflected off a target to the SPADs array, and the SPADs array had an FoV in the package to receiver the echo photons. The ranging sensor measures the time it takes for the emitted VCSEL pulse to travel back to the sensor, which determines the target distance. In addition, a bandpass filter with a center wavelength of 940 nm was used to cut down the background noise. The sensor operates in two types of output modes: (1) histogram raw data mode: output raw data of histogram, and (2) ranging mode: output distance data of ranging.

**Figure 2:**
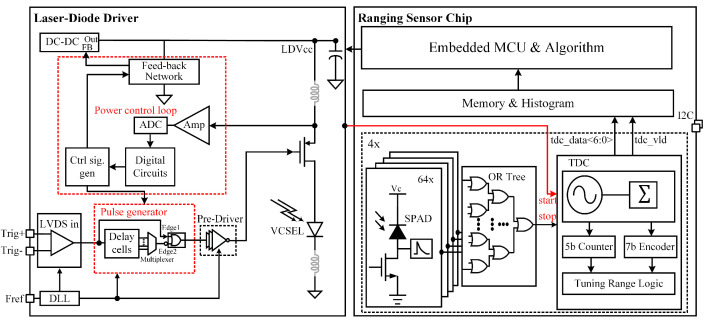
The ranging system with a pulsed VCSEL driver and SPAD-based sensor circuit implementation.

**Figure 3:**
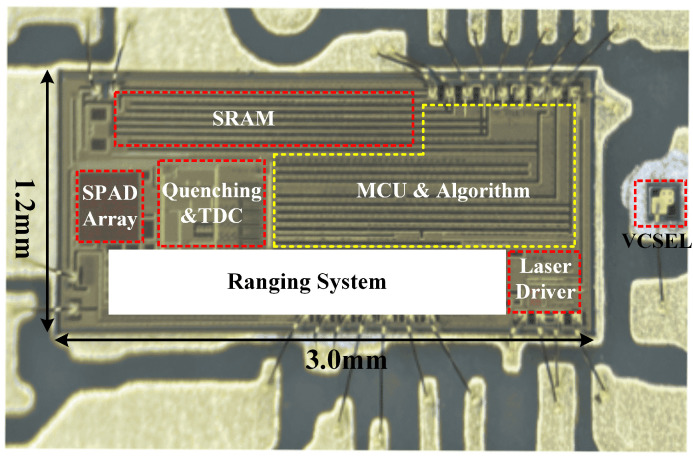
The micrograph of the ranging sensor chip.

The ranging sensor chip was fabricated in the 0.13 µm CMOS technology. The chip size is 1.2 mm × 3.0 mm. The chip consisted of a pulsed VCSEL driver and SPAD-based sensor, as shown in Figure 2. In the driver part, a programmable narrow pulse is generated on a chip via the pulse generator, and the first edge of the pulse (Edge1) comes directly from the external trigger, while the second edge (Edge2) is generated by properly delaying the trigger signal with a delay-cell line. The pulse delay is selected via the digital circuit. The output current of the driver is controlled by adjusting the laser driver voltage (LDVcc), which comes from a DC–DC regulator for which the feedback network is controlled by the digital circuit. A separate pulsed VCSEL driver was described by us in [15]. In the sensor part, a 16 × 16 SPADs array with quenching circuits was used, and each SPAD could be independently masked or binned to macro-pixel via OR Tree, four TDCs, a histogram builder that outputs the raw data of the histogram via I2C bus, and a firmware built-in MCU to implement the best profile-matched filter [16]. Figure 3 shows a micrograph of the ranging sensor chip.

## 3. Defining the Best Profile Matched Filter

**Figure 4:**
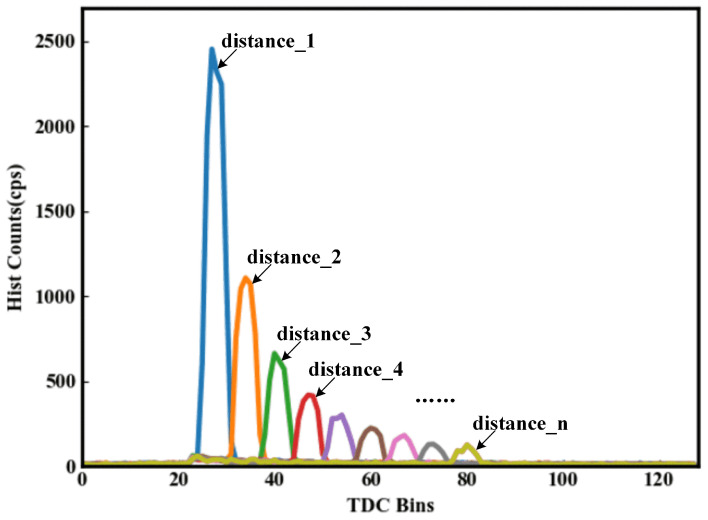
The histogram data distribution at different distances.

To find the best matched filter for our ranging system requires making our ranging system work in histogram raw data mode. A fully automated track was used to position the target at different distances for ranging, and we collected the raw data of the histogram at different distances. Figure 4 shows the raw data of the histogram at different distances. The laser ran at a repetition frequency of 1 MHz, with a 1 ns pulse width and an average power of 20 mW; the integration time of the ranging system was set to 30 ms; and the target reflectivity was 18%. We can see that in Figure 4, the SNR gradually decreases as the distance to the target object increases.

**Figure 5:**
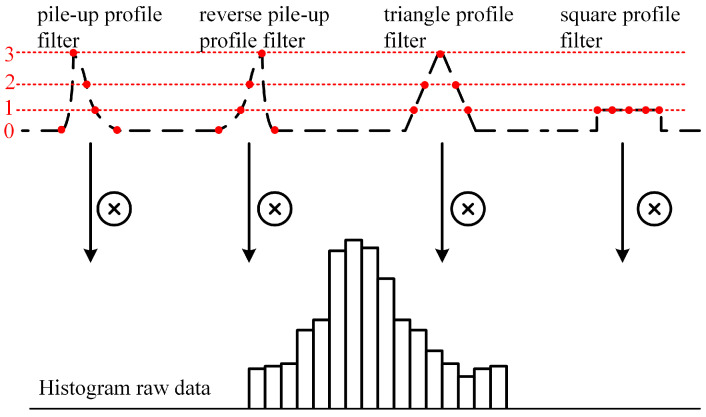
The processing of the different types of matched filters.

We take the raw data of the histogram and process it through a set of matched filters to find a matched filter with the lowest range jitter. In this work, we define four types of profile filters (pile-up, reverse pile-up, triangle, and square) to process the raw histogram data. The processing of the different types of matched filters is shown in Figure 5. The filter operation is performed by convolving the histogram with the filter kernel, $K_{fil}$, and the histogram processing method can be evaluated by using Equation (1) as follows.

(1) $$h\left(m\right)=\sum_{i=0}^{i<n}h_{raw}\left(m-i\right)*K_{fil}\left(i\right)$$

where h(m) is the *m*th filtered histogram bin, *m* is defined in the range of 0 to 127, *n* is the length of the filter kernel $K_{fil}$, *i* is the summation of the bins index, and $h_{raw}$ denotes the unprocessed bin values.

**Figure 6:**
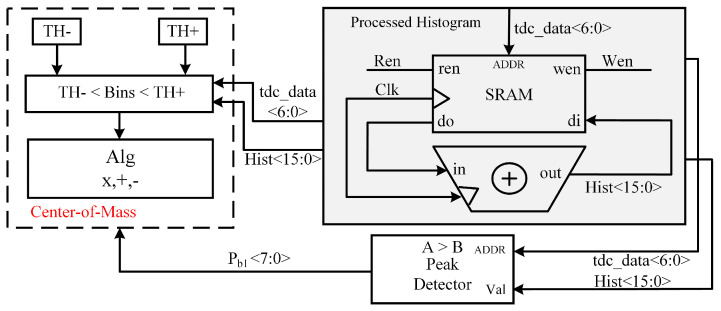
The implementation of the CoM algorithm.

We started with a single-point ranging calculation in order to characterize the precision of the different profile-matched filters in this ranging system. We defined precision as the standard deviation of the ranging system, and collected 200 sets of histogram data for each target distance. After the filtering process, the standard deviations of different filters at different distances are calculated using a CoM algorithm [17]. Figure 6 shows the implementation of the CoM algorithm. The processed histogram data output 7 bits of tdc_data and 16 bits of Hist, the tdc_data as the histogram bins value, and the Hist as the histogram count value of each histogram bin. The output values of the processed histogram are input into a peak detector circuit, and used to calculate the maximum peak bin as $P_{b1}$. We set a window of $P_{b1}$ with the width of full-width at half-maximum (FWHM) optical pulses. FWHM represents the FWHM of VCSEL. At this point, we have to map the 7-bit processed histogram data to the incoming TDC bin values (tdc_data), which have passed through the window such that Equation (2) holds. The low threshold value of the window is expressed by Equation (3), and the high threshold value of the window is expressed by Equation (4).

(2) $$TH_{-}<=TDC\_Bins<=TH_{+}$$

(3) $$TH_{-}=P_{b1}-\frac{FWHM}{2}$$

(4) $$TH_{+}=P_{b1}+\frac{FWHM}{2}$$

The range distance can be evaluated from the following CoM algorithm of Equation (5), where *h* represents the processed histogram bin counts, and *b* is the median of the background light counts. We perform 200 measurements for each distance to evaluate the range jitter.

(5) $$d=\frac{\sum_{t=TH_{-}}^{t=TH_{+}}t*max\left(0,h-b\right)}{\sum_{t=TH_{-}}^{t=TH_{+}}max\left(0,h-b\right)}$$

As we know the signal photon distribution within the laser pulse width, we can define the length of the filter kernel, $K_{fil}$, the same as the laser pulse width, and make the laser driver emit light with a pulse width of 1 ns. The TDC resolution is 500 ps in this ranging system, the jitter of the full system is approximately 1 ns, and the width of the matched filter is defined as 5. We designed the filter kernels of pile-up, reverse pile-up, triangle, and square, which are defined in Equations (6)–(9), respectively. The filter kernel is quantified by the filter profile in Figure 5.

(6) $$K_{fil\_pile-up}=\left\{0,3,2,1,0\right\}$$

(7) $$K_{fil\_reversepile-up}=\left\{0,1,2,3,0\right\}$$

(8) $$K_{fil\_triangle}=\left\{1,2,3,2,1\right\}$$

(9) $$K_{fil\_square}=\left\{1,1,1,1,1\right\}$$

**Figure 7:**
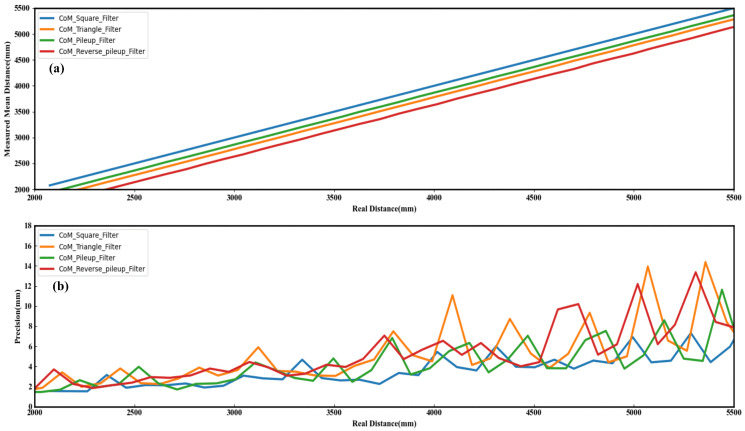
The results of 200 measurements with different profile-matched filters: (**a**) Mean distance. (**b**) Standard deviation.

Figure 7a shows the mean distance of 200 measurements with different profiles of matched filters, where the measured mean distance of the square profile filter is closer to the real distance. Figure 7b shows the standard deviation of 200 measurements with different profile-matched filters. There are 200 sets of histogram data for each distance. After each set of histogram data are processed by different profile-matched filters, the distance is calculated and the precision is evaluated using the CoM algorithm. The precision results of different profile-matched filters are compared with the ranging system. The depth precision of the square profile filter is less than 7 mm over the whole range, the depth precision of the triangle profile filter is less than 15 mm over the whole range, the square profile filter has the least jitter of ranging, and the triangle profile filter has the most jitter of ranging. In this ranging system, the square profile filter offers the best range precision, and with the above analysis, we implemented the square profile filter into the MCU core.

**Figure 8:**
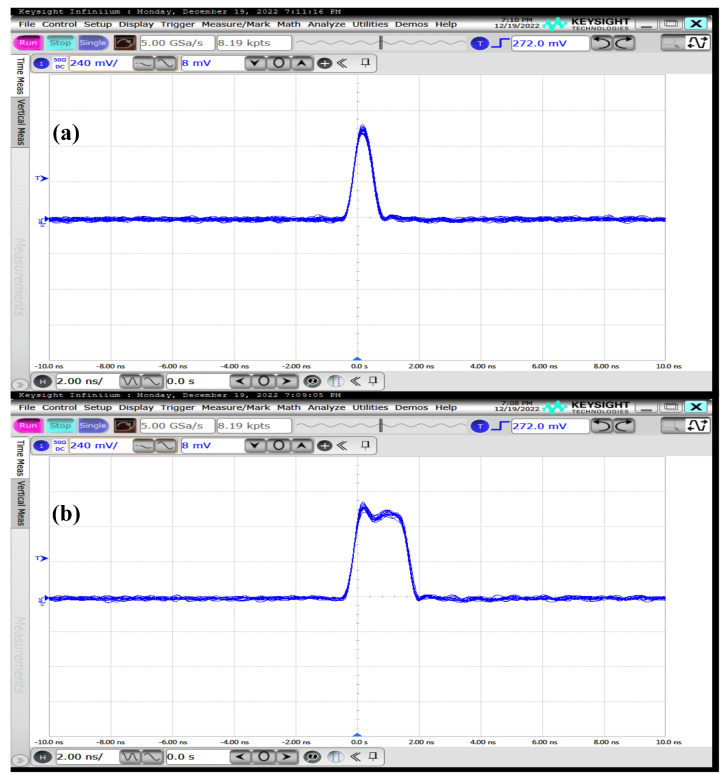
Different laser pulse widths: (**a**) Laser pulse width of 1 ns. (**b**) Laser pulse width of 2 ns.

Adjusting the pulse width of the laser also affects the jitter of the range measurement [18]. For lasers with different pulse widths, different lengths of filter kernels need to be designed to match the pulse width of the laser. Figure 8a shows a laser pulse width of 1 ns, and Figure 8b shows a laser pulse width of 2 ns. For different laser pulse widths, we designed square profile filter kernels of different lengths to process the raw histogram data. The filter kernels of 1 ns laser pulse width are defined in Equation (9), and the filter kernels of 2 ns laser pulse width are defined in Equation (10). The TDC resolution is 500 ps in this ranging system, and the jitter of the full system is approximately 1 ns. We need to set two elements to include the jitter of the whole system. If the laser pulse width is 1 ns, we need to set two elements to include the pulse width of the laser, and if the laser pulse width is 2 ns, we need to set four elements to include the pulse width of the laser. To satisfy the requirement of histogram distribution centrosymmetry, we set the length of the filter kernel to five for a 1 ns laser pulse width, and set the length of the filter kernel to seven for a 2 ns laser pulse width.

(10) $$K_{fil\_square\_2ns}=\left\{1,1,1,1,1,1,1\right\}$$

**Figure 9:**
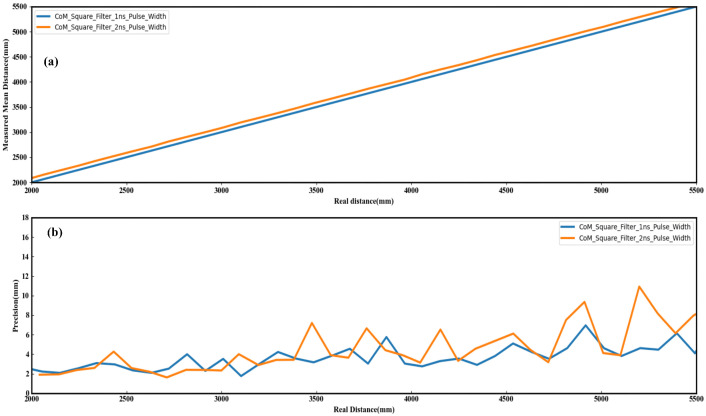
The results of 200 measurements with different laser pulse widths: (**a**) Mean distance. (**b**) Standard deviation.

The precision results of different widths of laser pulse are compared with the ranging system. Figure 9a shows the mean distance of 200 measurements with different laser pulse widths; for a laser pulse of 1 ns, the measured mean distance is closer to the real distance. Figure 9b shows the standard deviation of 200 measurements with different lengths of square profile-matched filter kernels. The depth precision is less than 6 mm over the whole range for a 1 ns laser pulse width, and the depth precision is less than 12 mm over the whole range for a 2 ns laser pulse width. With a 1 ns laser pulse width, the square profile filter has the least jitter of ranging. The narrower the pulse width of the laser, the more concentrated the distribution of the photon signals in the laser pulse, and the lower the jitter of ranging.

## 4. The Best Matched Filter and Ranging Results Implemented in the MCU Core

### 4.1. The Best Matched Filter Implemented in the MCU Core

**Figure 10:**
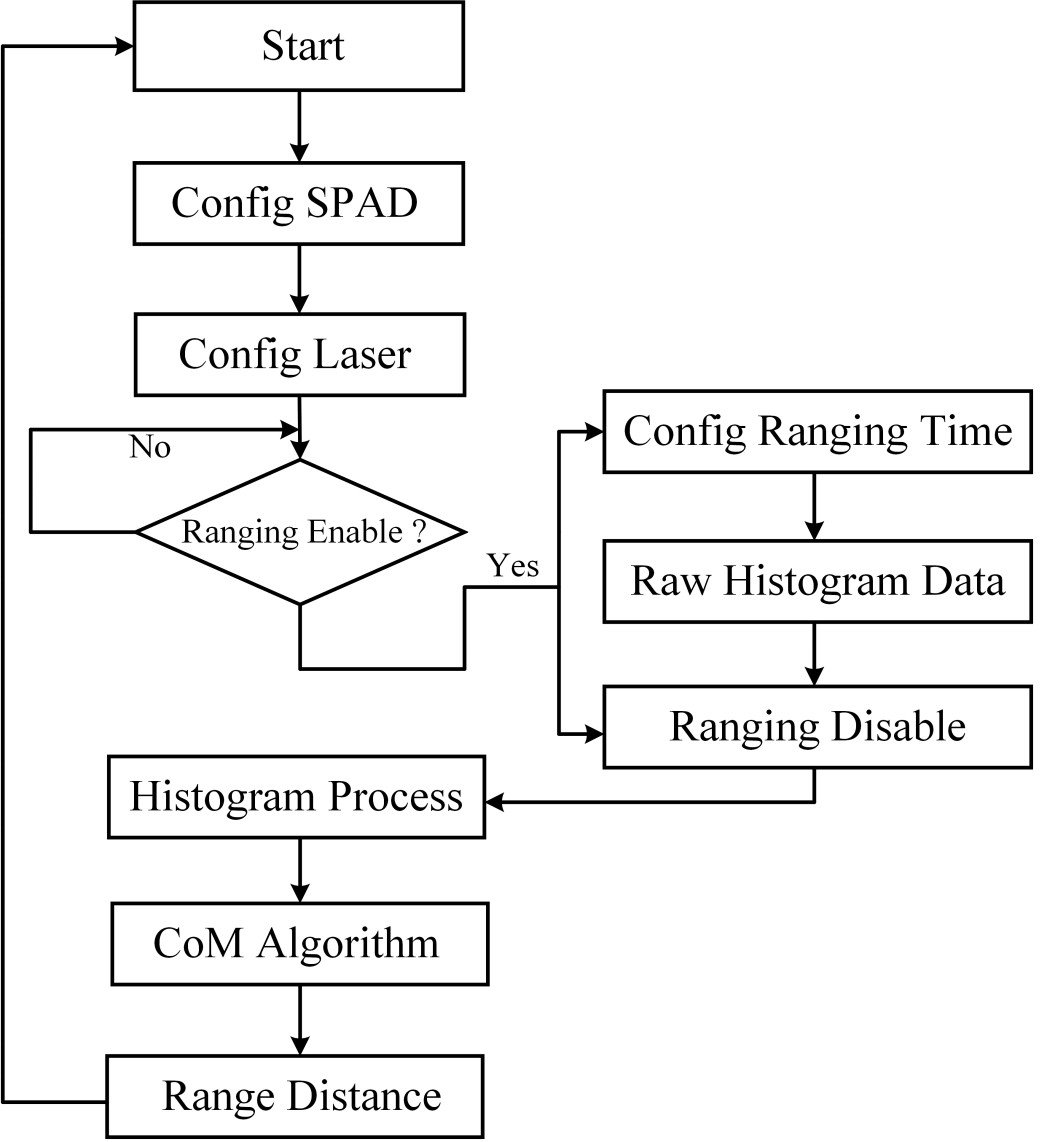
The best profile-matched filter implemented in the MCU core.

In this ranging SoC system, we use an MCU core to implement different profile-matched filters. Figure 10 shows the best profile-matched filter (square) implemented in the MCU core. Before each ranging, a hot-pixel checking algorithm (Config SPAD) is implemented to disable the hot pixels that exceed the high dark count rate (DCR) threshold, and the VCSEL is configured to emit at a pulse width of 1 ns, an optical power of 20 mW, and a frequency of 1 MHz. After the initial configuration is complete, we wait for the MCU to enable ranging, configure the time of ranging, and perform data collection for the raw histogram. The best profile-matched filter kernel is implemented in the MCU to process the raw histogram data. A CoM algorithm is implemented using the MCU to extract the final distance.

**Figure 11:**
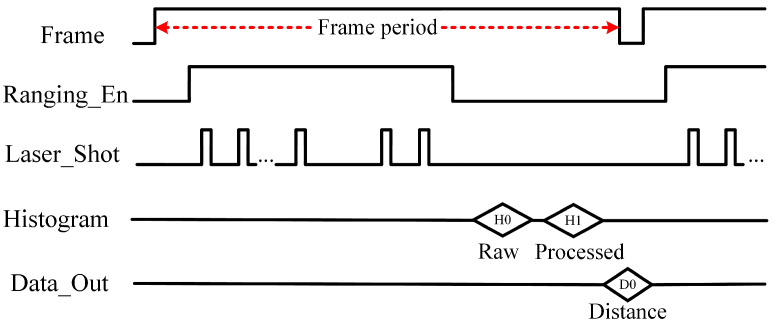
Timing diagrams of the proposed ranging system.

The timing diagram of the readout circuit is shown in Figure 11. At the beginning of each frame, the bias voltage circuit regulates the SPADs cathode voltage to a predefined value, preparing for effective signal detection. A Ranging_En starts the VCSEL emit laser pulses according the initial configuration to generate the timestamps. After the ranging period, the timestamps are collected as a histogram. The best profile-matched filter kernel is implemented in MCU to process the histogram, and a corresponding CoM algorithm is used for distance calculation [19]. The ranging distance is read out in serial-transmission mode.

### 4.2. The Ranging Results Measurement

**Figure 12:**
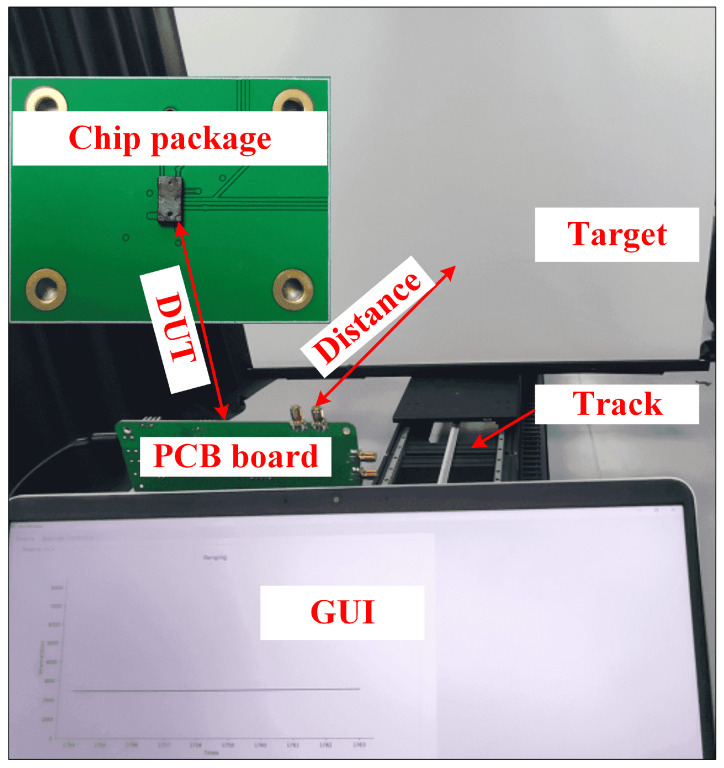
Ranging measurement setup showing how the VCSEL and sensor are packaged.

We started with a single-point range measurement in order to characterize the precision of the dToF ranging system. Figure 12 shows the setup for ranging measurements: a fully automated track that can position the target at different distances, a test-printed circuit board (PCB) board, and a GUI interface for displaying the measured distances. The VCSEL and the sensor are packaged into one module without any focusing lens. The laser is powered with 20 mW on average, 1 ns pulse width runs are carried out at a repetition frequency of 1 MHz, and the ranging sensor runs at 100 frames per second. Photons reflected from targets with different reflectivity are received by the ranging sensor. The distance from the target to the ranging sensor is calculated and the standard deviation can be obtained by measuring each distance 200 times.

**Figure 13:**
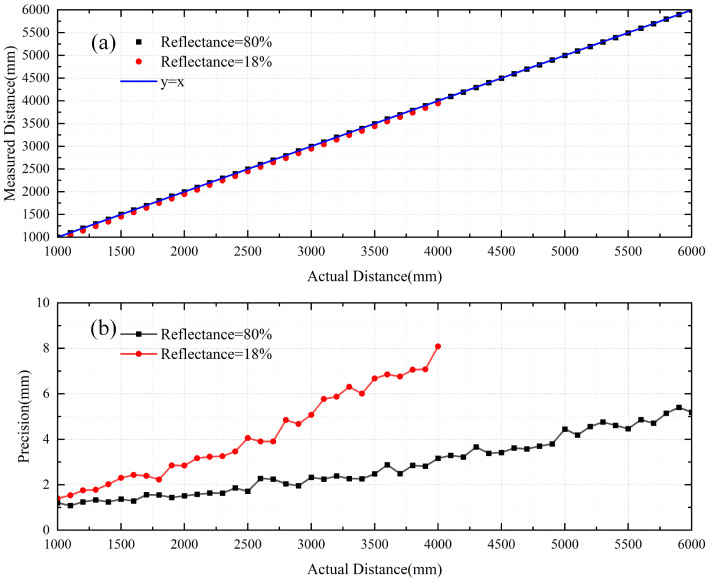
The linearity and depth precision vs. the actual distance larger than 1000 mm with different target reflectivity under indoor light conditions. (**a**) Linearity. (**b**) Depth precision.

According to the previous analysis, in the MCU core, we built in the best profile-matched filter (square) for depth precision calculation. The laser was powered with 20 mW, and 1 ns pulse width runs were carried out at a repetition frequency of 1 MHz. The measurement was conducted from 1000 mm to 6000 mm. In this system, for each distance, 200 measurements were implemented, processed, and then averaged to find the estimated distance value; the precision is the standard deviation of the measurement [20]. Figure 13a,b show the corresponding linearity and depth precision vs. the actual distance with different target reflectivity under indoor light conditions. The dToF system achieves a precision better than 5 mm over the whole range of 6 m with 80% target reflectivity, and with a precision better than 8 mm at a distance within 4 m with 18% target reflectivity.

A comparison of our system with the state-of-the-art ranging sensor systems is given in Table 1. Our ranging sensor has a depth precision better than 5 mm over the whole range of 6 m with 80% target reflectivity, and a precision better than 8 mm at a distance within 4 m with 18% reflectivity. Our ranging system achieves high-range precision at 6 m by using the best matched filter built in the MCU. The ranging system can be programmed with MCU cores, for use in other range applications. In [6], the maximum range is 3 m with 1.6 mm range precision, whereas in [20], the maximum range is 2 m with 2.3 mm range precision. Compared to the reference system [6,20], our system can achieve a longer range and a higher range precision of 5 mm at 6 m, and our system operates at a light wavelength of 940 nm to meet the needs of class-1 laser eye safety [21].

## 5. Conclusions

In LiDAR systems, ranging scenarios are complex and need to achieve long-range, high-precision detection. In different ranging scenarios, such as different reflectivity and different profiles of reflective surfaces, these changes can distort the distribution of the histogram; when the distribution of the histogram becomes distorted, it causes a large ranging error. In order to reduce the error caused by the distortion of the histogram distribution, we need to find a best matched filter. In this work, we propose a method to find the best matched filter to suit our ranging system, and the built-in MCU of our ranging system is able to implement different filter profiles to meet the needs of different ranging scenarios and different ranging precisions for LiDAR applications.

This work presents a dToF ranging sensor system-on-a-chip, consisting of a configurable SPAD array, a 940 nm VCSEL, a co-optimized laser driver, and an MCU core to implement the best matched filter. In this work, we propose a scheme to design filters of different profiles to process the raw histogram data, and find the best filter with the least distance jitter. Finally, the best profile filter was implemented in the MCU and used in a ranging system. The system had a precision of better than 5 mm over the whole range of 6 m with 80% target reflectivity, and a precision better than 8 mm at a distance within 4 m with 18% reflectivity. Longer-range laser ranging with higher precision can be expected in the future.

## Figures and Tables

**Table 1:** System performance summary and comparison table.

Performances	This Work	[6]	[20]
Technology	**0.13 µm**	0.15 µm	0.16 µm
No. of pixels	**16 × 16**	50 × 40	40 × 10
Laser wavelength	**940 nm**	650 nm	670 nm
Target reflectivity	**80%, 18%**	90%	80%
Max range	**6 m@80%, 4 m@18%**	3 m	2 m
Max dis. precision	**5 mm@80%, 8 mm@18%**	1.6 mm	2.3 mm
Standard deviation	**2.2 mm@80%@3 m**	1.6 mm@3 m	2.3 mm@2 m

## Data Availability

Not applicable.
